# Heart lessons

**DOI:** 10.5935/1678-9741.20150050

**Published:** 2015

**Authors:** Domingo M. Braile

**Affiliations:** 1 Editor-in-Chief BJVCS/RBCCV

The last 12 months have been of deep losses for the Brazilian cardiovascular surgery. Distinguished and competent professionals, fundamental to the consolidation and advancement of the specialty in our country have left us, after serving brilliantly their career in this world. On July 10, 2014, Dr. Geraldo Verginelli died. On November 12, 2014, Dr. Raul Correia Rabelo left our living and just two days later, we had the death of Dr. Adib Domingos Jatene. In 2015, we had three more losses: on February 21, Dr. Edgar San Juan died (see Memorial, written by Dr. Vera Piccardi on page 395), on May 26, Dr. Marcos Vinicius Ferraz Arruda has lost the battle to cancer, the same occurred on the 5^th^ of July with Dr. João Alberto Roso.

But we can not just lament these happenings. We must look into the achievements of these cardiac surgeons, draw lessons from their teachings and apply them in our day-to-day, not only in the professional field, but also in personal life. Thanks to the dedication of the pioneers in times when our specialty still crawled, without stopping, let alone giving up in the face of obstacles, making their work and creativity and managing to overcome difficulties, it was possible to put the Brazilian cardiac surgery in the prominent place it occupies today in the world.

Their example will be forever and the best way to honor them is with great dedication, whether at work, whether in research, so that the problems we face today with the crisis befalling the country and affects everyone, decreasing financial resources, do not take the courage nor the will to fulfill our role as best as possible. And these teachers gave us real lessons of the heart that we must always have them saved, especially in times where the situation appears adverse. Dr. Adib said: "I am against this story to tell: - I don't do because they don't give me conditions. If you are able to do you create the conditions." We can learn from these true heart lessons!

Brazilian Journal of Cardiovascular Surgery (BJCVS) emerged from this dream, and if today is an internationally recognized journal, it owes much to the pioneers. In 2016, we will complete 30 years of uninterrupted movement and we intend to celebrate this important date with a series of events and activities, held in conjunction with the Brazilian Society of Cardiovascular Surgery (BSCVS), which we'll disclose in the coming issues.

We have been constantly concerned about the BJCVS meet the requirements of databases. An example was the language shift to English and increasing the number of issues of 4 to 6 per year, adoption of standard XML and DOI, among other examples. In July, at the suggestion of Scielo, we will adopt the CC-BY license instead of the CC-BY-NC, previously used. This maximizes the open access and dissemination of science, ensuring that the credit of the authors is properly attributed^[1]^. If any author who has submitted their study to the BJCVS understands that such change generates conflict, I ask to contact the Editorial Board.

## ABEC course

In order to keep us updated regarding the news of scientific communication, I participated, along with the Executive Editor, Ricardo Brandau, and the Editorial Assistant Camila Safadi of the XIII Scientific Publishing Course, promoted by the Brazilian Association of Scientific Editors (ABEC) of 25-27 June in Goiânia-GO. The topics discussed were of interest not only of publishers but also of authors and reviewers. The quality of the studies and the concern with ethics (which involves the issue of plagiarism) were issues largely discussed.

The speakers emphasized the increasing concern about the quality, which should be of authors, reviewers and editors, permeating the entire process of execution and submission of a manuscript from the survey data, text writing with accuracy, the properly review and approval, publishing and making available by the journal.

The role of all involved in this process is important. I high-light the reviewer's role, whose observations for the study to be improved are essential so that a quality science is published. At the meeting of ABEC ways to reward the work of revision were discussed. While the CAPES does not accept the suggestion to "reward" those who made a minimum number of evaluations score, each journal has sought alternatives. The BJCVS publishes every edition the list of reviewers who evaluated the studies that are part of that issue (in this issue the listing is on page 401). Also, after each review, the system generates a certificate that can be printed or stored electronically.

## Checking of manuscripts

Also in relation to the manuscripts, the importance of the Letter to the Editor was discussed, written in the submission of the study. Without ceasing to be a succinct, it should contain a brief presentation of the study. This will facilitate the Editor's decision-making with regard to the choice of reviewers accustomed to the topic, streamlining the flow.

I would also like to request that the authors, when submitting the manuscript make a prior review not only of the content and form, but also the other information contained in the study, such as author names, name of institution, address, etc. There have been cases of errors in these items, which are only perceived by those who submitted after approval and even publication of the article. In addition to the time spent to send the correction to the site, the biggest problem in the case of wrong names, is the need to request the correction by the Crossref so that the DOI does not bring misinformation and to put an Erratum. I emphasize the need for collaboration for these details either at the time of submission as in checking the PDF before publication, so that these problems are avoided.

## Globalization x misconduct

Another aspect that has been addressed in discussions of scientific publications and was discussed in Goiania is the misconduct issue, which involves several nuances, such as fraud and plagiarism. As much care that publishers take, either through a careful reading of the manuscript or using programs that detect plagiarism, there is always the risk, and if the article is published, it brings many problems to the authors, the editor and the journal.

The Internet, which cut distances, facilitated scientific communication and, in recent years, the number of article submissions had a big jump, as well as the requirements of the institutions and development agencies. This creates a true "race to publish" in which ethical principles, which should guide the science, are not always respected. In 2011, the article "Pressure to Publish How Globalization and Technology are Increasing Misconduct in Scholarly Research"^[2]^, a report ("white paper") available from iThenticated, addressed the issue, showing the problems and concluded that it is necessary that beyond the prevention of misconduct, mechanisms should be created to ensure intellectual property.

BJCVS reaffirms its repudiation of political plagiarism - which appears in Rules for Authors (http://www.rbccv.org.br/page/6) - and misconduct and will continue to support actions aiming to combat this nefarious practice in science.

## CME

The articles with CME in this issue are: *"Hydrocortisone supresses inflammatory activity of metalloproteinase - 8 in carotid plaque"* (page 295), *"Chronotropic incompetence in Chagas disease: effectiveness of blended sensor (volume/minute and accelerometer)"* (page 311), *"Mitral annulus morphologic and functional analysis using real time tridimensional echocardiography in patients submitted to unsupported mitral valve repair"* (page 325) , and *"Effect of remote ischemic postconditioning in inflammatory changes of the lung parenchyma of rats submitted to ischemia and reperfusion"* (page 353).

I highlight, as always, the importance of CME to update knowledge, noting that the test is worth 0.5 linear points in the SBCCV Proof of Title.

My warmest regards,

Domingo M Braile Editor-in-Chief BJVCS/RBCCV
